# Supplementary material for: OTUD6B regulates KIFC1-dependent centrosome clustering and breast cancer cell survival
Source: EMBO Rep. 2025 Jan 9;26(4):1003–35. doi: 10.1038/s44319-024-00361-w (PMC11850729; doi:10.1038/s44319-024-00361-w)
Supplement: Supplementary file 1 — Appendix [file 44319_2024_361_MOESM1_ESM.pdf]

APPENDIX

Table of Contents

Appendix Table S1. Summary of DUB screens. 2
Appendix Figure S1. Supplementary data for DUB siRNA library screens. 4
Appendix Figure S2. Examples for deconvolution of DUB siRNA pools from the screens revealing off-target effects. 5
Appendix Figure S3. OTUD6B copy number is associated with increased mRNA expression and protein levels in breast cancer samples. 6
Appendix Figure S4. JOSD2 is not frequently altered in breast cancer samples. 7
Appendix Figure S5. OTUD6B depletion decreases KIFC1 expression without altering its localisation. 9
Appendix Figure S6. OTUD6B localises to the nascent spindle in mitosis. 10
Appendix Figure S7. Expression and ubiquitination of KIFC1 during mitotic exit. 12
Appendix Figure S8. Supporting data for studies on OTUD6B depletion and knockout requirement for cell viability. 13

Appendix Table S1. Summary of DUB screens.

Colour coding as indicated in Fig 1B and Fig 1D.

DUB family	Number
USPs	55
OTU	15
JAMM	11
JOSEPHIN	4
UCH	4
Papain-like	1

	Western blotting screen for KIFC1	Imaging screen for multipolar mitoses
DUB	Log2 (KIFC1: gel mean & mean of controls)	Log2 (multipolar mitoses: mean of controls)
CYLD	0.34	0.55
PAN2	-0.69	0.47
USP1	-0.31	0.61
USP10	-1.07	0.67
USP11	1.11	0.57
USP12	-0.59	0.58
USP13	0.39	0.24
USP14	0.65	0.63
USP15	0.93	0.44
USP16	-0.09	0.52
USP17	0.52	-0.78
USP17L2	0.47	0.11
USP17L6P	-0.80	-0.17
USP18	1.30	0.19
USP19	-0.27	0.04
USP2	0.36	0.37
USP20	-0.41	0.51
USP21	0.04	0.56
USP22	-0.15	0.39
USP24	0.55	0.32
USP25	-0.08	0.07
USP26	-0.26	0.44
USP27X	-0.80	0.14
USP28	1.36	0.36
USP29	-0.25	0.08
USP3	-1.79	0.25
USP30	0.19	-0.50
USP31	0.38	0.33
USP32	0.33	0.63
USP33	-0.42	0.38
USP34	0.66	0.12
USP35	-0.96	0.61
USP36	0.11	-0.27
USP37	0.27	0.72
USP38	0.43	-0.58
USP39	-0.65	0.21
USP4	-0.21	0.67
USP40	0.62	-0.43
USP41	0.84	0.12
USP42	-0.04	-0.08
USP43	0.36	-0.26
USP44	0.90	0.55
USP45	0.37	-0.08

**USP46**	-0.68	0.25
**USP47**	0.31	0.03
**USP48**	-0.29	0.61
**USP49**	0.98	0.27
**USP5**	-0.40	-0.29
**USP50**	-0.73	0.71
**USP51**	-0.25	-0.38
**USP53**	-0.10	-0.36
**USP54**	-0.17	0.31
**USP6**	-0.40	0.39
**USP7**	-0.43	0.04
**USP8**	-0.30	-0.09
**USP9X**	-0.67	2.59
**USP9Y**	0.16	0.11
**USPL1**	N/A	0.21
**FAM105B**	-0.06	-0.53
**OTUB1**	-0.45	-0.39
**OTUB2**	0.78	0.56
**OTUD1**	-0.45	0.26
**OTUD3**	-0.01	-0.58
**OTUD4**	0.49	-0.08
**OTUD5**	0.63	0.09
**OTUD6A**	-0.08	0.28
**OTUD6B**	-1.78	1.01
**OTUD7A**	0.19	0.62
**OTUD7B**	0.11	0.10
**TNFAIP3**	-1.08	-0.49
**VCPIP1**	0.48	-0.33
**YOD1**	0.41	0.13
**ZRANB1**	-0.49	0.30
**BRCC3**	0.15	0.36
**COPS5**	-0.31	0.51
**COPS6**	-0.27	-0.53
**EIF3F**	0.09	0.30
**EIF3H**	0.04	-0.95
**MPND**	-0.12	-0.26
**MYSM1**	-0.46	-1.46
**PRPF8**	0.87	1.21
**PSMD14**	0.62	0.31
**PSMD7**	-0.15	-2.16
**STAMBP**	-1.02	-0.62
**STAMBPL1**	-0.34	0.27
**ATXN3**	0.46	-0.21
**ATXN3L**	-0.02	0.55
**JOSD1**	0.21	-0.53
**JOSD2**	-1.51	0.76
**BAP1**	-0.24	-0.61
**UCHL1**	-1.21	0.39
**UCHL3**	-0.01	0.06
**UCHL5**	-0.24	0.20
**C14orf28**	0.04	0.17

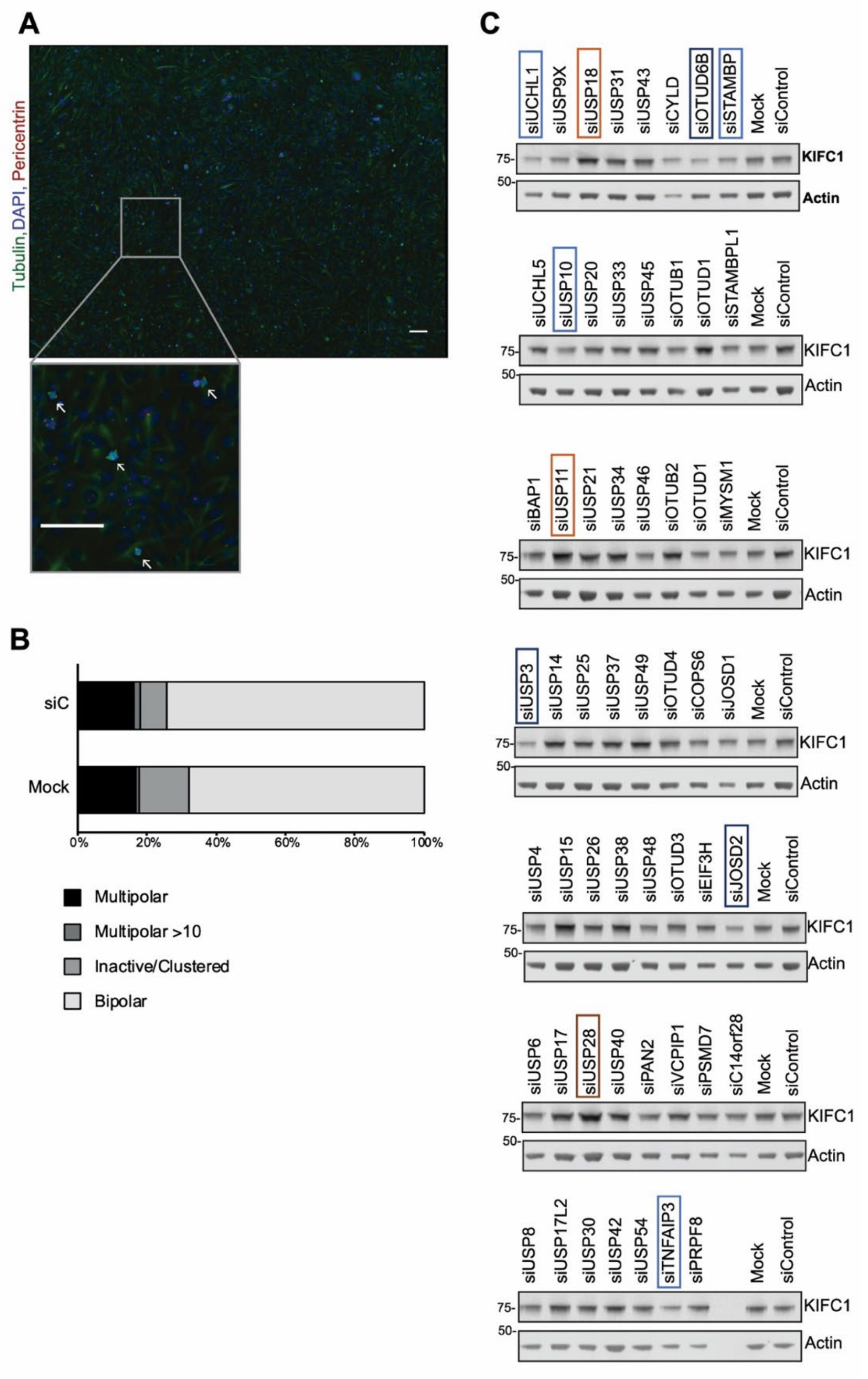

Appendix Figure S1. Supplementary data for DUB siRNA library screens.

A Example stitched image used to score multipolar spindles, scale bar 100 µm. **B** The range of multipolar spindle frequency in cells for the control conditions in the screen (mock and siC). **C** Extracts from the KIFC1 immunoblotting screen shown in **Fig 1**. Blue or orange borders indicate, respectively, DUBs whose depletion decreased or increased levels of KIFC1 by >2-fold.

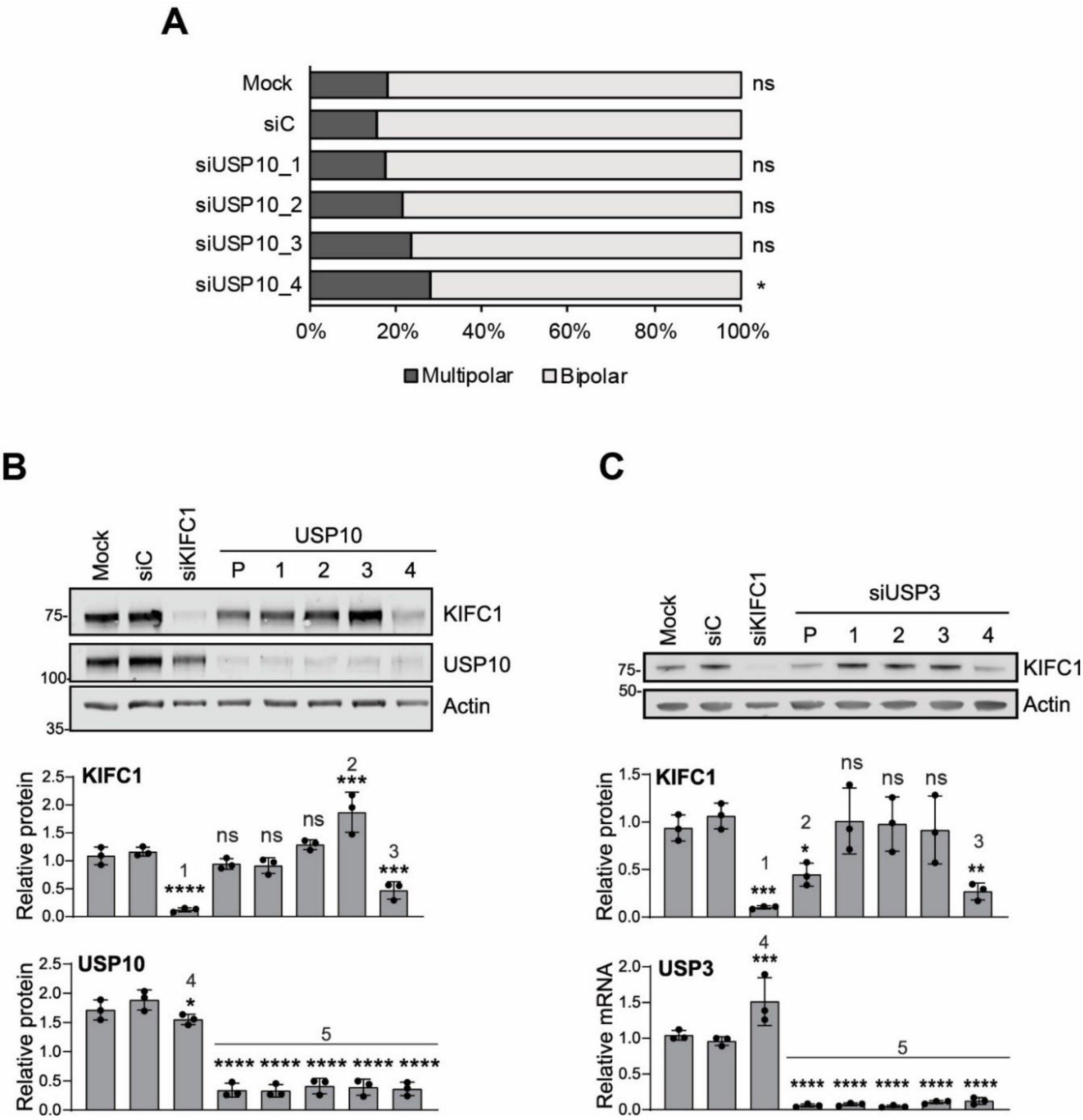

Appendix Figure S2. Examples for deconvolution of DUB siRNA pools from the screens revealing off-target effects.

A-B Although USP10 was a hit in both screens, transfection with 40 nM individual siRNAs suggest this is likely an off-target effect of siUSP10_4. Centrosome de-clustering (**A**); >50 metaphase cells scored per condition in n=3 biological replicates; ns, not significant, *P=0.0116 by one tailed Chi-square test compared to siC control. KIFC1 expression (**B**); protein expression normalised to actin and the mean of the controls; error bars SD of n=3 biological replicates; [1]****P≤0.0001, [2]***P=0.0006, [3]***P=0.0007, [4]*P=0.0382, [5]****P ≤ 0.0001, ns, not significant, compared to siC by one-way ANOVA with Dunnett post-hoc test. USP3 was the top hit in the KIFC1 screen, however deconvolution using individual USP3 siRNAs shows this is likely an off-target effect of siUSP3_4 (**C**). Protein expression normalised to actin and the mean of the controls; error bars SD of 3 biological replicates, [1]***P =0.0004, [2]*P =0.0187, [3]**P =0.0026, [4]***P=0.0004 [5]****P≤ 0.0001, ns, not significant, compared to siC by one-way ANOVA with Dunnett post-hoc test. Transfection with the 4 pooled siRNAs (P).

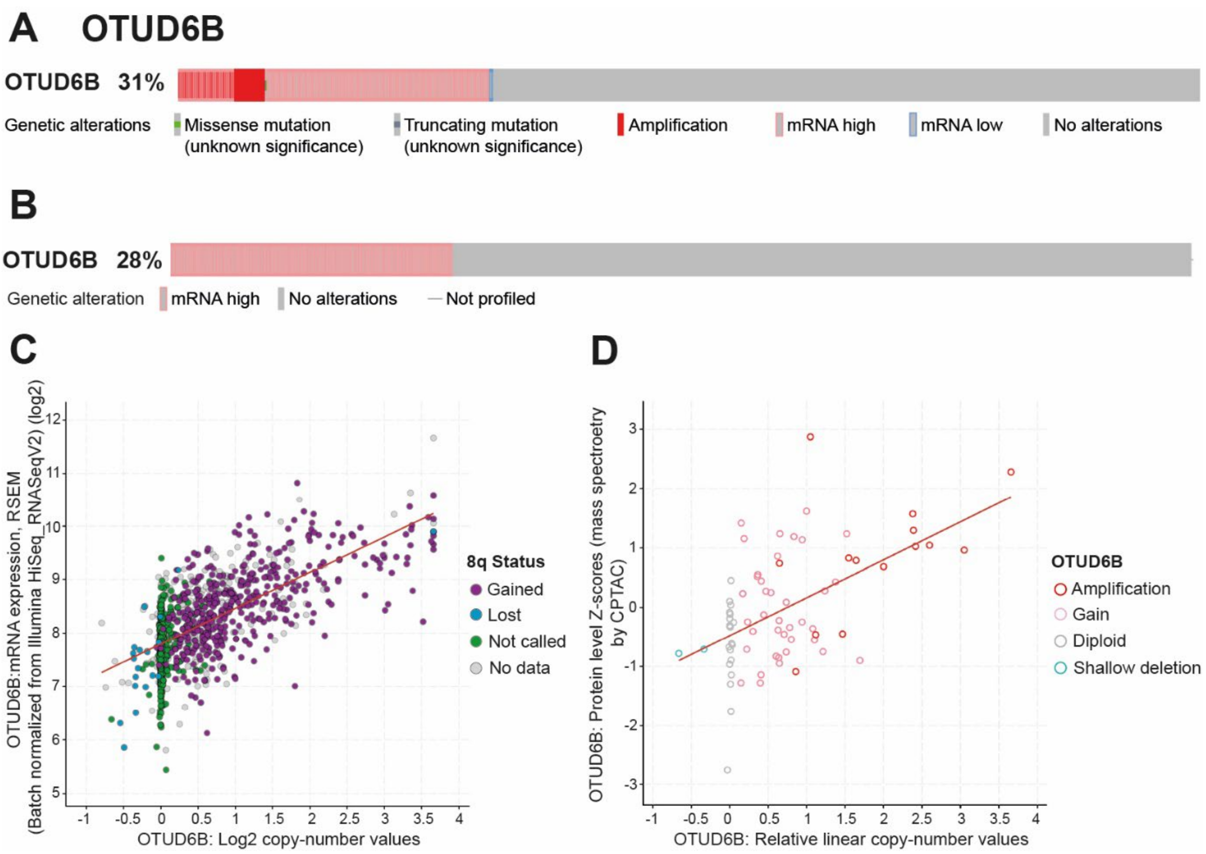

Appendix Figure S3. OTUD6B copy number is associated with increased mRNA expression and protein levels in breast cancer samples.

Summary of alterations in OTUD6B in the TCGA (n=1082) and CPTAC (n=105) TCGA PanCancer Atlas breast invasive carcinoma datasets; mRNA expression RNAseq, protein levels mass spectrometry. **A-B** Oncoprints summarising all genetic/mRNA alterations (**A**) and mRNA high (**B**) in TCGA dataset. **C-D** OTUD6B copy number amplification correlates with OTUD6B mRNA expression, Spearman correlation 0.65, P = 7.58 x 10^{-128} (**C**) and protein level, Spearman correlation 0.45, P = 6.136 x 10^{-5} (**D**).

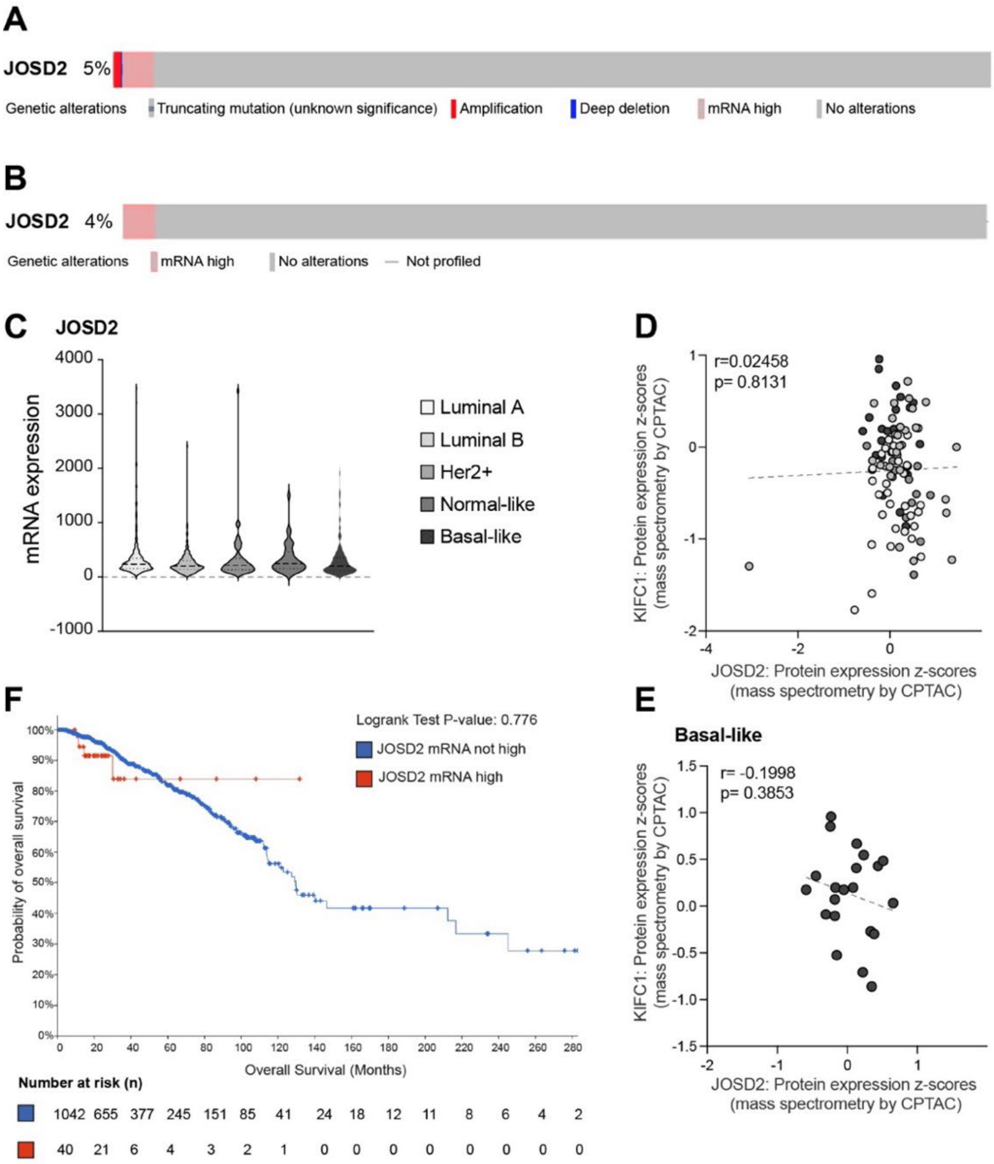

	Number of cases, Total	Number of Events	Median Months Overall (95% CI)
cases with JOSD2 mRNA not high	1042	147	129.57 (114.80 - 216.75)
cases with JOSD2 mRNA high	40	4	NA

Appendix Figure S4. JOSD2 is not frequently altered in breast cancer samples.

Summary of alterations for JOSD2 in the TCGA and CPTAC PanCancer Atlas breast invasive carcinoma datasets; mRNA expression by RNAseq, protein levels by mass spectrometry. **A-B** Oncoprints summarising genetic and mRNA expression alterations (**A**) or mRNA high (**B**) in TCGA dataset (n=994). **C** JOSD2 mRNA expression is not elevated in any breast cancer subtypes in the TCGA dataset (n=981). Patient data were stratified according to subtype and compared to Basal-like by Kruskal-Wallis with Dunn's multiple comparison test. **D-E** Scatter plots comparing JOSD2 and KIFC1 protein levels in CPTAC dataset (**D**, n=95) or in those samples classified as basal-like (**E**, n=21); one-tailed Pearson coefficient. **F** JOSD2 mRNA overexpression is not associated with prognosis. Kaplan-Meier estimate of overall survival for all breast cancer patients stratified by JOSD2 mRNA expression in the TCGA dataset; n=994, *P*=0.756, long rank test.

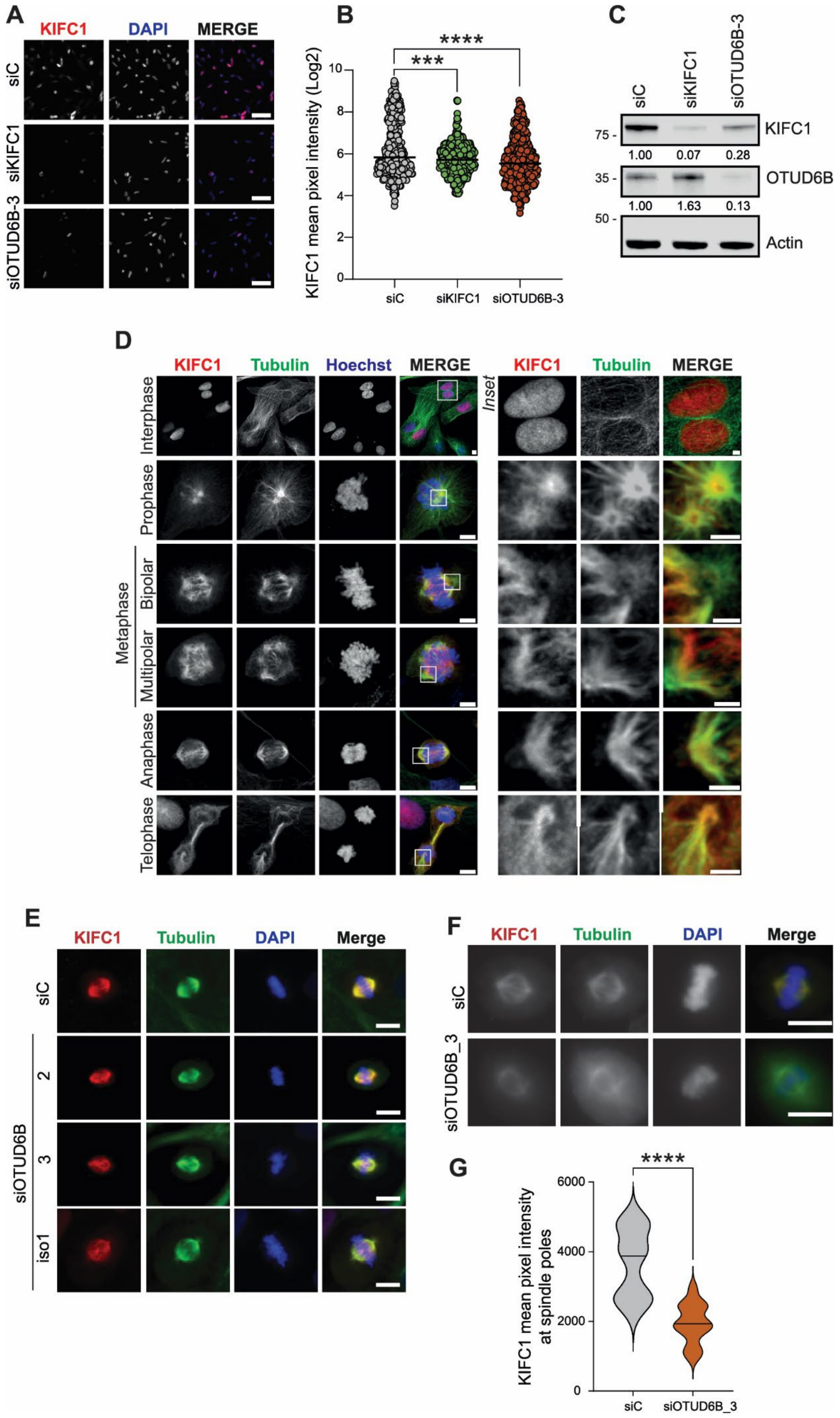
A
KIFC1
DAPI
MERGE
siC
siKIFC1
siOTUD6B-3
B
KIFC1 mean pixel intensity (Log2)
****
***
siC
siKIFC1
siOTUD6B-3
C
siC
siKIFC1
siOTUD6B-3
75 -
KIFC1
1.00 0.07 0.28
35 -
OTUD6B
1.00 1.63 0.13
50 -
Actin
D
KIFC1
Tubulin
Hoechst
MERGE
Inset
Interphase
Prophase
Metaphase
Bipolar
Multipolar
Anaphase
Telophase
E
KIFC1
Tubulin
DAPI
Merge
siC
siOTUD6B
2
3
iso1
F
siC
siOTUD6B_3
G
KIFC1 mean pixel intensity at spindle poles
****
siC
siOTUD6B_3

Appendix Figure S5. OTUD6B depletion decreases KIFC1 expression without altering its localisation.

A-C MDA-MB-231 cells were transfected with 10 nM siRNA for 72 h then stained for KIFC1 or DNA (DAPI). Representative example of images acquired on Nikon Eclipse Ti fluorescent microscope, CFI Plan Apochromat 40× N.A. 0.95, W.D. 0.14mm objective, scale bar 50 µm (**A**). Mean pixel intensity (Log2) scored for KIFC1 after background subtraction was measured for 500 cells per condition in n=1 biological replicate; ***P=0.0002, ****P≤0.0001 compared to siC by Kruskal-Wallis with Dunn's multiple comparison test (**B**). Immunoblot confirming siRNA depletion (**C**). **D** KIFC1 localisation changes through the cell cycle. MDA-MB-231 cells were stained for KIFC1, spindle (tubulin) and DNA (Hoechst) and presented as Z-stack projection; images taken using a Zeiss LSM800 confocal microscope; scale bar 5 µm. **E-G** OTUD6B depletion reduces KIFC1 at spindle poles. BT549 cells were transfected with 10 nM siRNA for 72 h then stained for KIFC1, spindle (tubulin) and DNA (DAPI). Representative images acquired on Nikon Eclipse Ti fluorescent microscope, for >25 mitotic cells per condition in n=3 biological replicates; scale bar 10 µm (**E**). Example sets of images for siC or siOTUD6B_3 treated cells acquired with identical KIFC1 channel settings for quantification, scale bar 10 µm (**F**). In FIJI/ImageJ a 3µm diameter circular region of interest was added over individual spindle poles, KIFC1 mean intensity measured for ≥14 poles per condition, ****P≤0.0001 by unpaired t-test (**G**).

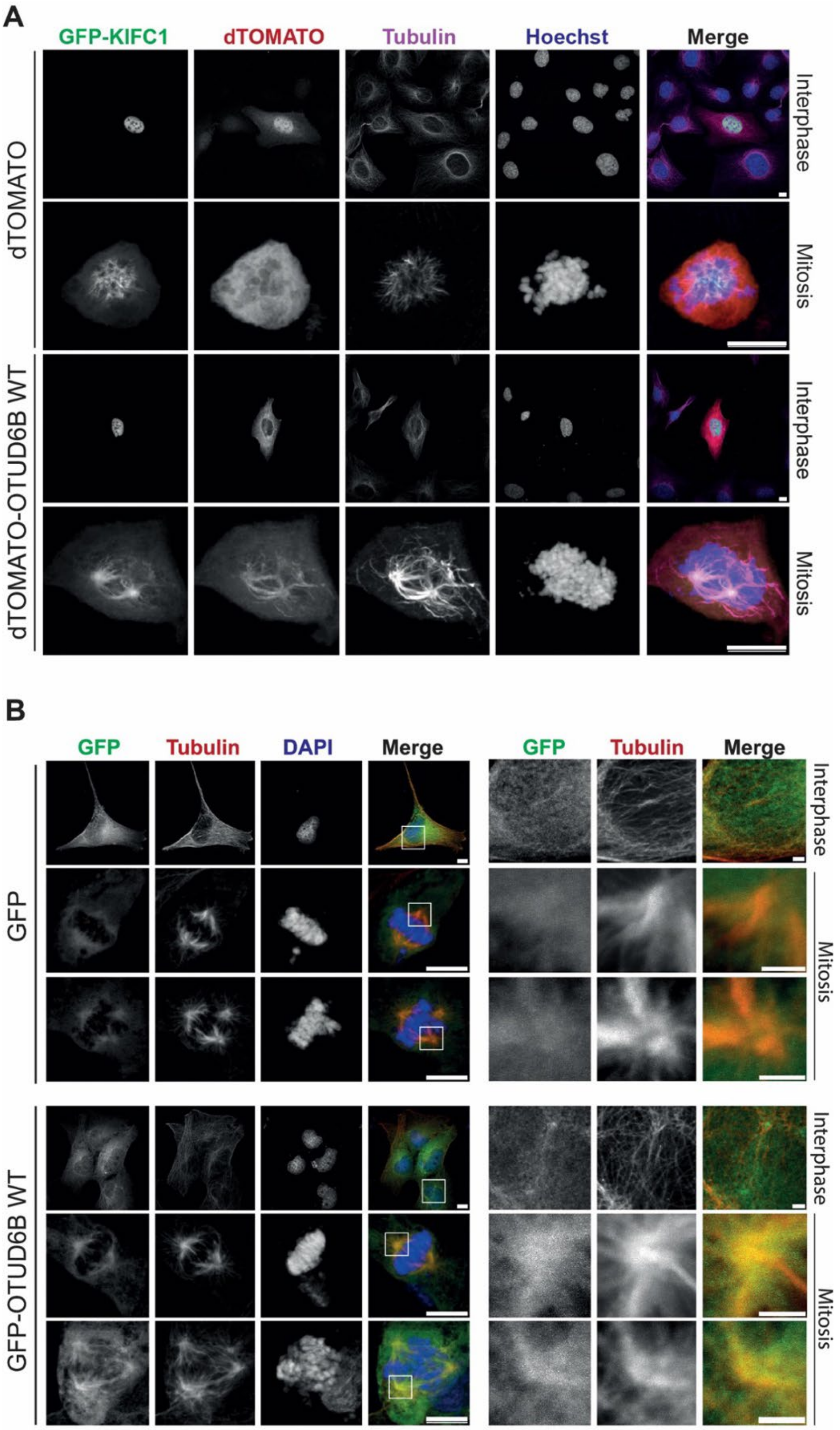

Appendix Figure S6. OTUD6B localises to the nascent spindle in mitosis.

U2OS (**A**) or BT549 (**B**) cells were seeded on a glass coverslip 24 h before transfection with the specified plasmids. 48 h after transfection, cells were fixed and stained for tubulin (red **A,** purple **B**) and DNA using Hoechst I (**A**) or DAPI (**B**) (blue). Images taken on a Zeiss LSM800 confocal microscope, using Plan Apochromat 63X NA 1.4 OIL objective are presented as Z-stack projection. Scale bar 10 µm, scale bar inset 2 µm (**A**); scale bar 5 µm (**B**).

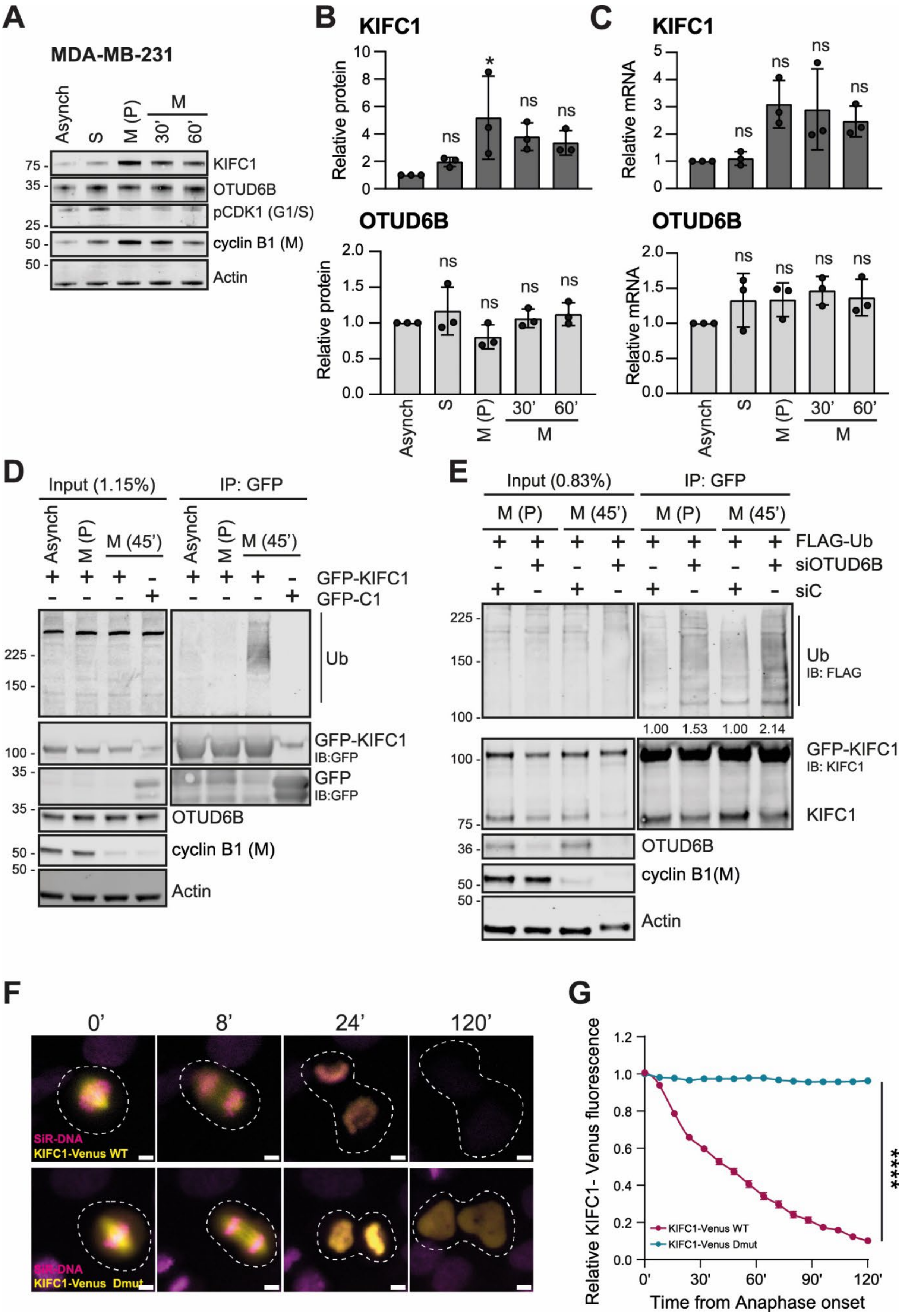
A
MDA-MB-231
Asynch
S
M (P)
M
30'
60'
KIFC1
OTUD6B
pCDK1 (G1/S)
cyclin B1 (M)
Actin
B
KIFC1
Relative protein
OTUD6B
C
KIFC1
Relative mRNA
OTUD6B
ns
*
D
Input (1.15%)
IP: GFP
M (45')
GFP-KIFC1
GFP-C1
Ub
IB:GFP
GFP
E
Input (0.83%)
FLAG-Ub
siOTUD6B
siC
Ub
IB: FLAG
1.00
1.53
1.00
2.14
IB: KIFC1
cyclin B1(M)
F
0'
8'
24'
120'
SiR-DNA
KIFC1-Venus WT
KIFC1-Venus Dmut
G
Relative KIFC1- Venus fluorescence
Time from Anaphase onset
****

Appendix Figure S7. Expression and ubiquitination of KIFC1 during mitotic exit.

A-C KIFC1 levels are regulated through the cell cycle in MDA-MB-231. Cells were synchronised using thymidine/STLC/ZM447439 and parallel samples lysed for immunoblotting or RNA extracted for qRT-PCR. Representative immunoblot (**A**) and KIFC1 protein level normalised to asynchronous cells (**B**). KIFC1 mRNA expression determined by qRT-PCR normalised to ACTB and relative to asynchronous cells (**C**). Mean of n=3 biological replicates, **P*=0.0205, ns, not significant, compared to asynchronous cells by Kruskal-Wallis with Dunn's multiple comparison test (KIFC1) and One-Way ANOVA with Dunnett's multiple comparison test (OTUD6B). **D-E** U2OS cells were transfected with plasmids (48 h) and siRNAs (10 nM, 72 h), synchronised at prometaphase (M-P) or collected 45 min into mitotic exit (M-45'), then immunoprecipitated with GFP-nanobeads, n = 1 biological replicate. Endogenous ubiquitylation of GFP-KIFC1 is increased during mitotic exit (**D**). OTUD6B depletion increases KIFC1 ubiquitylation at prometaphase and at 45 min into mitotic exit (**E**). **F-G** Timecourse imaging of KIFC1-Venus degradation during mitotic exit. U2OS cells were transfected with plasmids (48 h), synchronised at G1/S by double-thymidine block, released in medium supplemented with 50 nM SiR-DNA and imaged with a Nikon Eclipse Ti fluorescent microscope, CFI Plan Apochromat 40× N.A. 0.95, W.D. 0.14mm objective. Representative images of dividing cells at different time points after anaphase onset (**F**), scale bar 10 µm, dotted line indicates mitotic cell. KIFC1-Venus levels in individual mitotic cells were quantified, an *in vivo* degradation curve was plotted as a function of anaphase onset (**G**); mean indicated, >40 individual mitotic cells scored per condition in n=2 biological replicates, error bars SEM, *****P*≤ 0.0001 between KIFC1-Venus WT and Dmut at 120' by Mann-Whitney test.

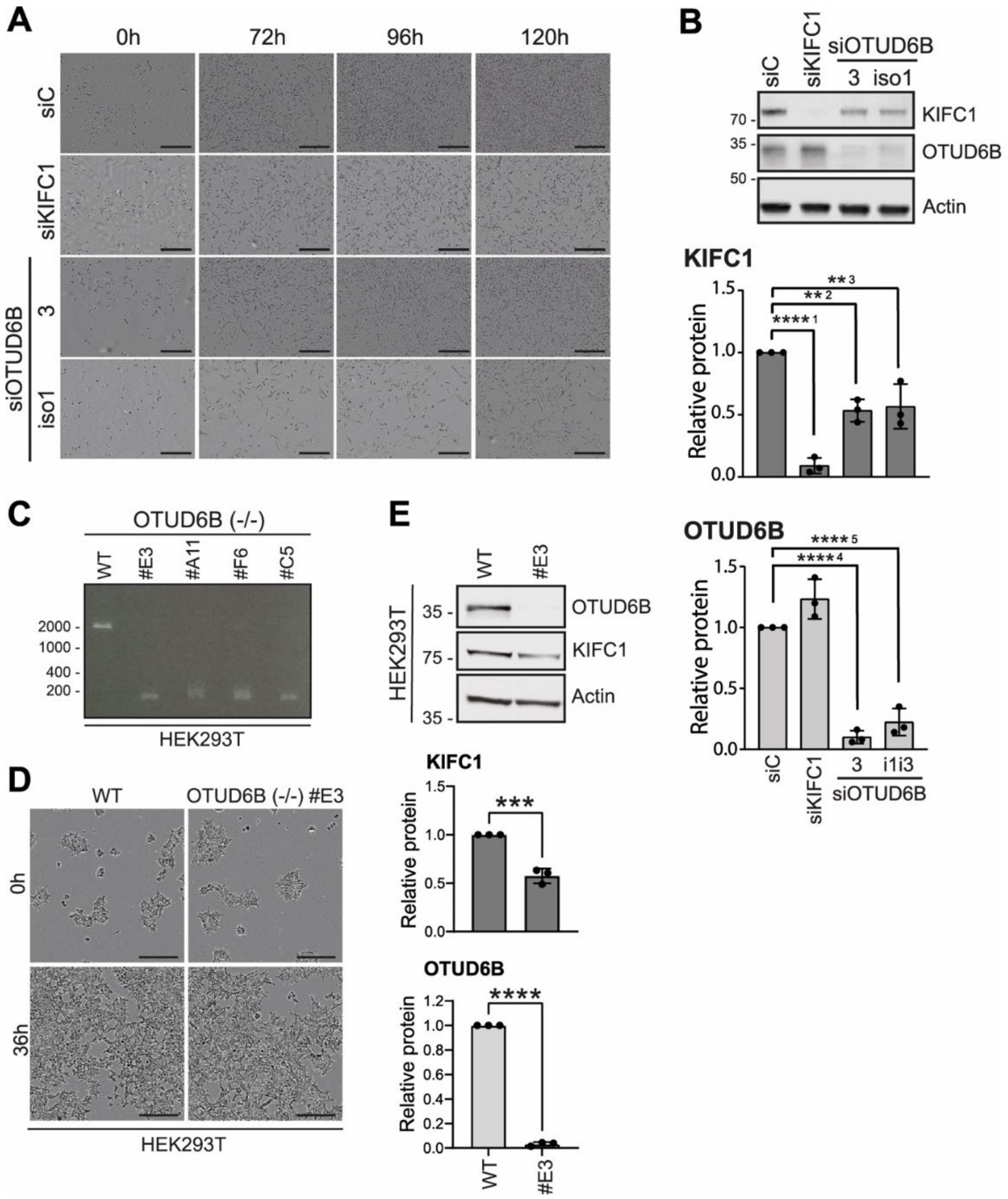

Appendix Figure S8. Supporting data for studies on OTUD6B depletion and knockout requirement for cell viability.

A-B OTUD6B depletion affects viability of TNBC cells. MDA-MB-231 cells were transfected with 10 nM siRNA and confluence monitored for 120 h by live cell imaging, for the experiment shown in **Fig 7B**. Representative images captured using an IncucyteS3 (**A**), scale bar 200 µm. Confirmation of depletion by immunoblotting (**B**); n=3 biological replicates, [1]****P≤0.0001, [2]**P=0.0016, [3]**P=0.0026, [4]****P≤0.0001, [5]****P≤0.0001 compared to siC by One-Way ANOVA with Dunnett's multiple comparison test. **C-E** OTUD6B CRISPR/Cas9 knockout of OTUD6B in HEK293T cells. Genomic DNA was extracted from wild type and OTUD6B CRISPR edited HEK293T clones. End point PCR shows full length (1972 bp) OTUD6B in WT and the single ~190 bp band generated by CRISPR editing with the sgRNA pair, indicating homozygous knockout clones (**C**). 4 weeks post-transfection with sgRNAs, CRISPR edited HEK293T (clone E3) were imaged for 36 h (Incucyte) alongside parental HEK293T cells to confirm cell growth (**D**); scale bar 200 µm. Representative immunoblot of HEK293T *OTUD6B*$^{+/+}$ and *OTUD6B*$^{-/-}$ clone E3 showing quantification of KIFC1 and OTUD6B normalised on actin and relative to HEK293T *OTUD6B*$^{+/+}$ (**E**); n=3 biological replicates, ***P=0.0006, ****P≤ 0.0001 by one-sample t-test relative to HEK293T *OTUD6B*$^{+/+}$ cells.
