# Supplementary material for: OTUD6B regulates KIFC1-dependent centrosome clustering and breast cancer cell survival
Source: EMBO Rep. 2025 Jan 9;26(4):1003–35. doi: 10.1038/s44319-024-00361-w (PMC11850729; doi:10.1038/s44319-024-00361-w)
Supplement: Supplementary file 9 — Source data Fig. 1 [file 44319_2024_361_MOESM9_ESM.zip › Figure 1/EMBOR-2023-58722_Figure 1A/DUB screen for centrosome clustering.docx]

**DUB screen for centrosome clustering: Protocol**

**Aim: Does knockdown of any of the DUB family result in centrosome de-clustering?** The DUB siRNA library will be transfected into BT549 cells in 2 96 well plates. After knockdown cells will be stained with pericentin, tubulin and DAPI to allow identification of normal and multipolar mitotic spindles by microscopy. Images of all wells will be taken and then % of multipolar spindles in each scored.

Reverse transfections of Qiagen pool plate into BT549 cells

There are 92 DUB siRNA pools in Qiagen pool plate.

siRNAs were transposed from pool plate to 2 screening plates, filling outer wells with PBS to prevent evaporation and minimise edge effects. This allowed for 6 Mock and 6 siC wells per plate.

siRNA conc:

1. 40nM: 0.5ul siRNA in 125ul

Qiagen pool plate (copy 4) stock is 10uM.

siC (Qiagen Allstars) stock is 20uM, dilute small aliquot 1:2 in RNAse-free water to 10uM.

**Reverse Transfection**

1. Dilute RNAiMax 1 in 20 with OptiMEM.

Need 3ul well x 106 wells = 318ul total (15.9ul RNAiMax + 302.1ul OptiMEM).

1. Add 21.75 ul OptiMEM per well
2. Add 0.5ul siRNA per well
3. Add 3ul diluted RNAiMax per well.
4. Mix well- gently knock the plate against hand several times whilst rotating the plate- ensure bottom of each well in now covered with liquid.
5. Incubate at room temp for 20 min.
6. Meanwhile trypsinise BT549, p12, cells and resuspend in normal full media (RPMI+10%FCS+ 40ul Inuslin/500ml bottle):

Resuspend at 8x10^4^/ml. Add 100ul to each well i.e. 8000 cells.

1. Leave overnight then replace media with 200ul fresh, full.

**72 hours after transfection, fix cells for IF staining**

1. Gently pipette off media.
2. Add 100ul 4%PFA+0.1%TritonX100 in TBS, pre-warmed to 37C, for 15mins.
3. Remove PFA and add 100ul 1% BSA in TBS for 30mins to block.
4. Remove and add 40ul primary antibody mix* and incubate 2 hour RT.
5. Remove and add 40ul secondary antibody mix** and incubate 1 hour RT.
6. Remove and wash 1xTBS.
7. Add DAPI at 1ug/ml in TBS for 10mins.
8. Remove and wash 1XTBS
9. Remove and store in 100ul TBS at 4C until ready for imaging.

*Anti-pericentrin, rabbit, Abcam, 1:1000 +anti-tubulin DM1A, mouse, Sigma, 1:1000 in TBS.

**Alexa 594 anti-rabbit 1:500 + Alexa 488 anti-mouse 1:500 in TBS.

**Imaging and analysis**

Cells imaged on Nikon with 6x6 20x fields of view (stitched as one large image) taken per well. This looked to give>50 mitotic cells/well.

- Files saved as indicated in plate layout, i.e. A1, A2 etc where A1 refers to well A1 in siRNA library (not to well number it is in in the sreening plate).
- Images opened one at a time, in order i.e. A1 first, H10 last.
- Scoring done WITHOUT referring to library plate list.
- Once all scoring complete, raw data recorded in excel, then in new sheet, Raw data aligned to library list of DUBs for further analysis.

All prometaphase and metaphase cells in image analysed and classed as one of the following five categroies:

1. **Bipolar**: either normal (2centrosomes) or clustered centrosomes, bipolar spindle, aligned DNA.
2. **Multipolar**: de-clustered centrosomes, more than 2 spindle poles, misaligned DNA.
3. **>10 centrosomes multipolar**.
4. **Bipolar, inactivated centrosomes, all cemtrosomes togethe**r: Where there is a bipolar spindle but extra centrosomes are present but do not nucleate microtubules and therefore do not effect DNA alignment. In this case, all extra, inactiavted centrosomes appear together.
5. **Bipolar, inactivated centrosomes, centrosomes not together:** Where there is a bipolar spindle but extra centrosomes are present but do not nucleate microtubules and therefore do not effect DNA alignment. In this case, all extra, inactiavted centrosomes are not together.

Also, any unusual morphologies, a high degree of apoptotic cells or other comments noted.
