# Supplementary material for: OTUD6B regulates KIFC1-dependent centrosome clustering and breast cancer cell survival
Source: EMBO Rep. 2025 Jan 9;26(4):1003–35. doi: 10.1038/s44319-024-00361-w (PMC11850729; doi:10.1038/s44319-024-00361-w)
Supplement: Supplementary file 9 — Source data Fig. 1 [file 44319_2024_361_MOESM9_ESM.zip › Figure 1/EMBOR-2023-58722_Figure 1A/ReadMe File for Main Figure 1A_C Multipolar mitosis DUB microscopy screen.docx]

**ReadMe File for Main Figure 1A-C: Multipolar mitosis DUB microscopy screen**

- The other word file in this folder describes the protocol for how the screen was performed, imaged and analysed.
- The folder contains the original .nd2 files and the cropped .tif files of the example phenotype images shown in 1A.
