# Supplementary figures and images for: OTUD6B regulates KIFC1-dependent centrosome clustering and breast cancer cell survival
Source: EMBO Rep. 2025 Jan 9;26(4):1003–35. doi: 10.1038/s44319-024-00361-w (PMC11850729; doi:10.1038/s44319-024-00361-w)

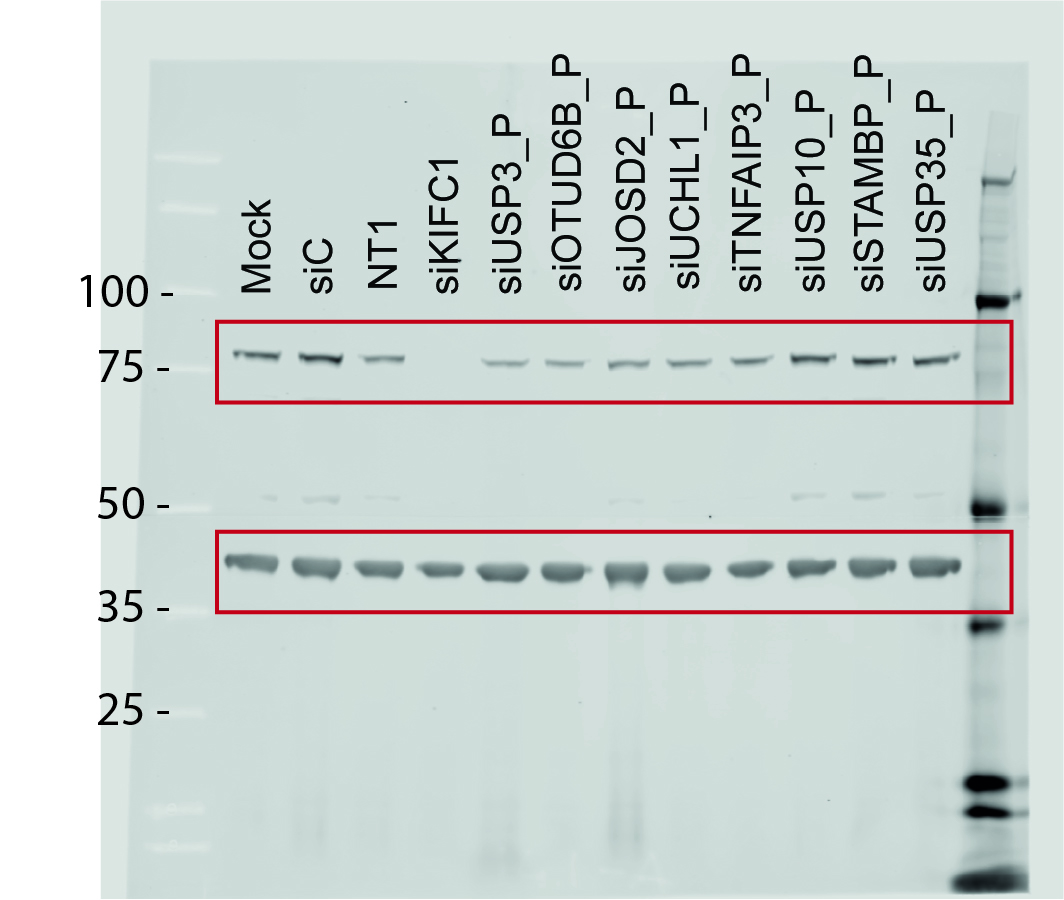

Supplement: Supplementary file 9 — Source data Fig. 1 [file 44319_2024_361_MOESM9_ESM.zip › Figure 1/EMBOR-2023-58722_Figure 1E/Figure 1E - WB images.jpg]

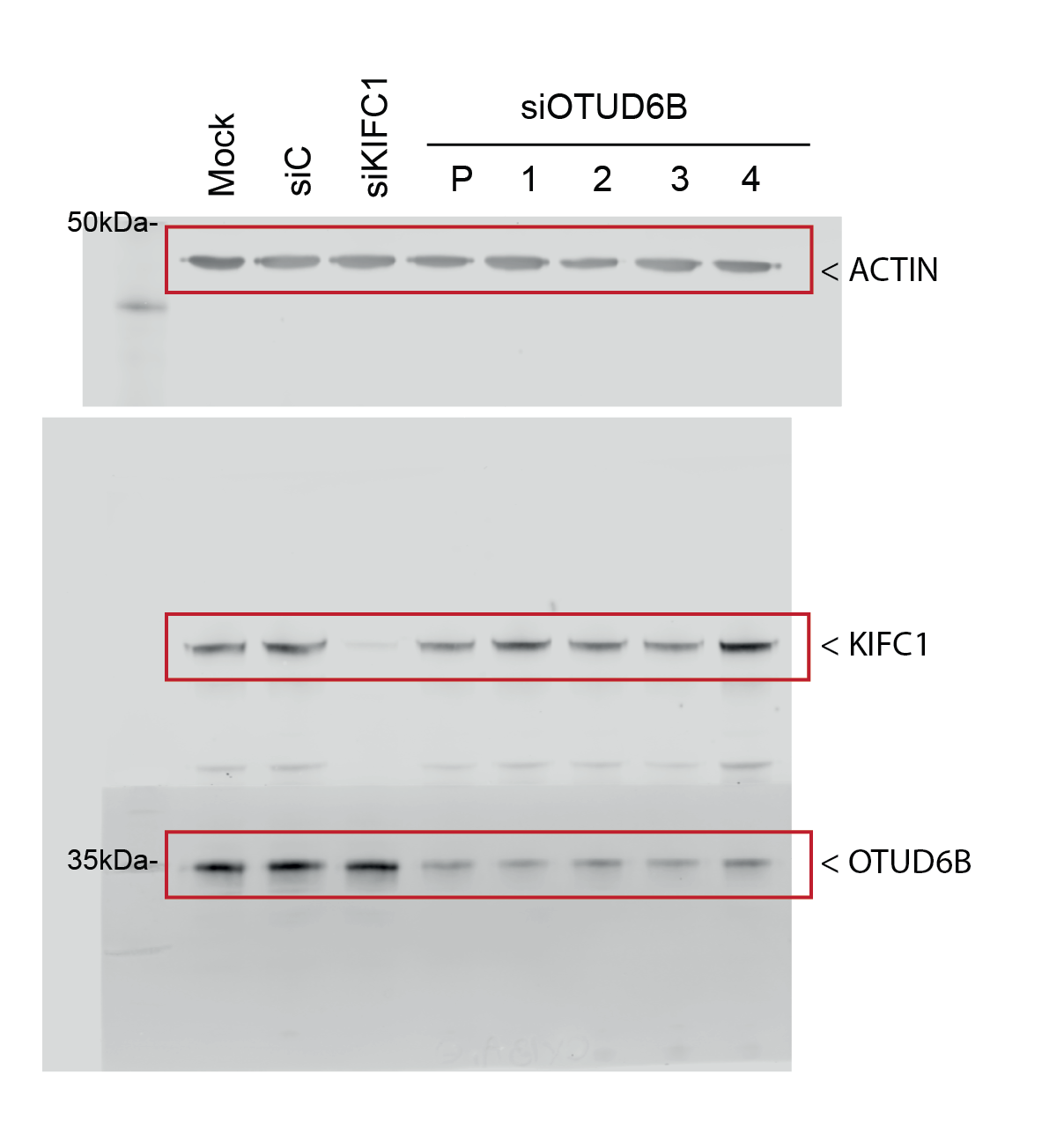

Supplement: Supplementary file 10 — Source data Fig. 2 [file 44319_2024_361_MOESM10_ESM.zip › Figure 2/EMBOR-2023-58722_Figure 2C/EMBOR-2023-58722_Figure 2C - Deconvolution of siRNA pools for OTUD6B.png]

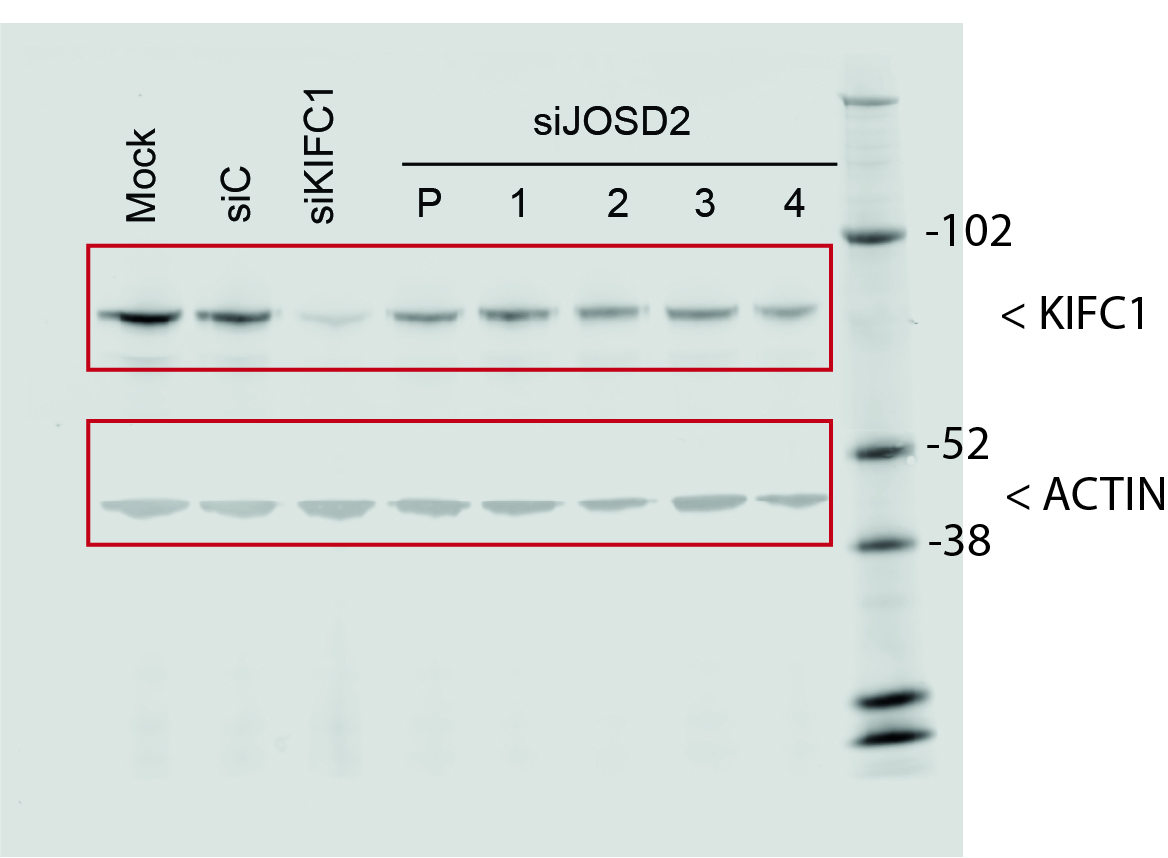

Supplement: Supplementary file 10 — Source data Fig. 2 [file 44319_2024_361_MOESM10_ESM.zip › Figure 2/EMBOR-2023-58722_Figure 2D/Figure 2D - Deconvolution of siRNA pool for JOSD2.jpg]

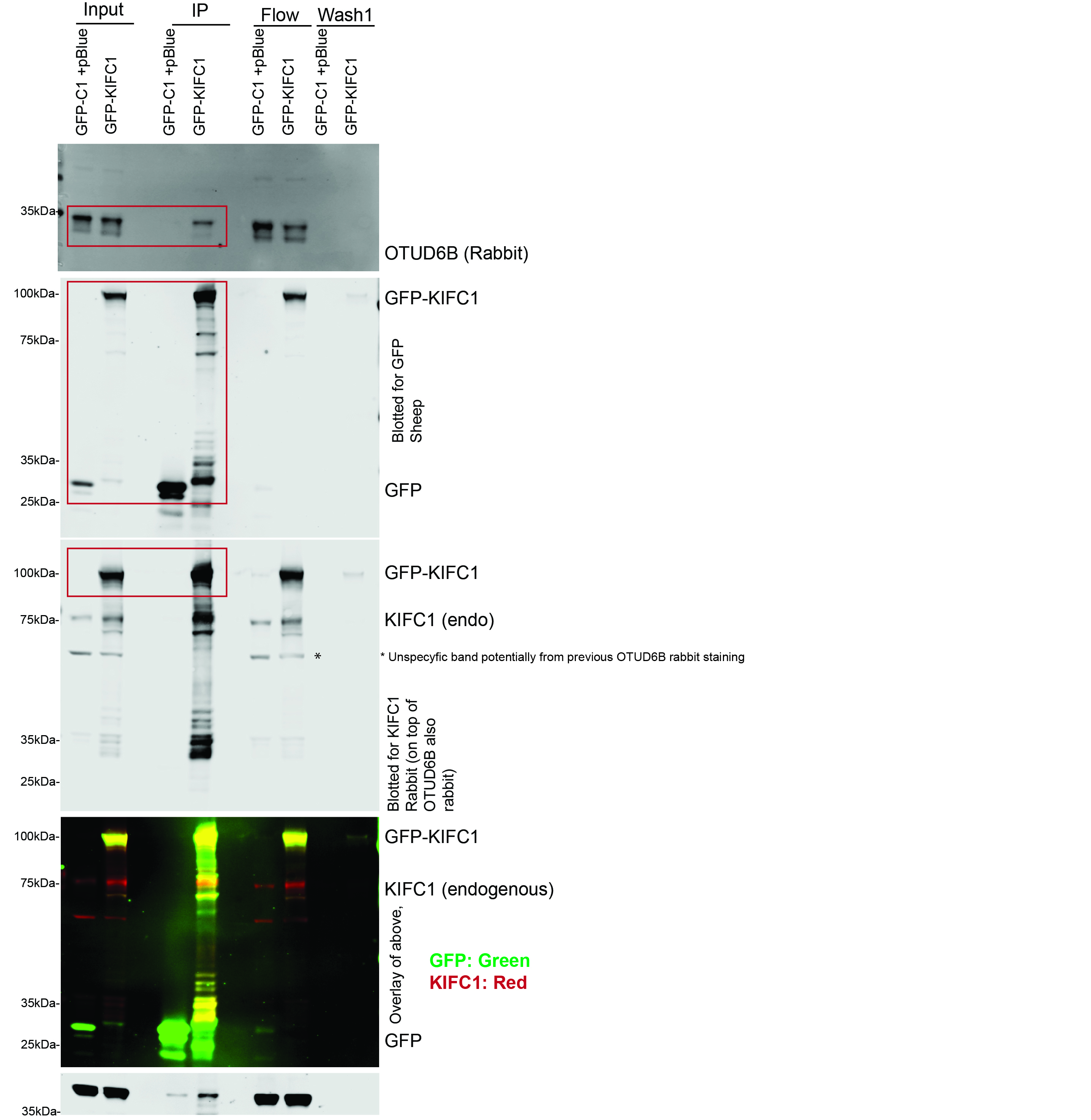

Supplement: Supplementary file 12 — Source data Fig. 4 [file 44319_2024_361_MOESM12_ESM.zip › Figure 4/EMBOR-2023-58722__Figure 4F/Figure 4F.jpg]

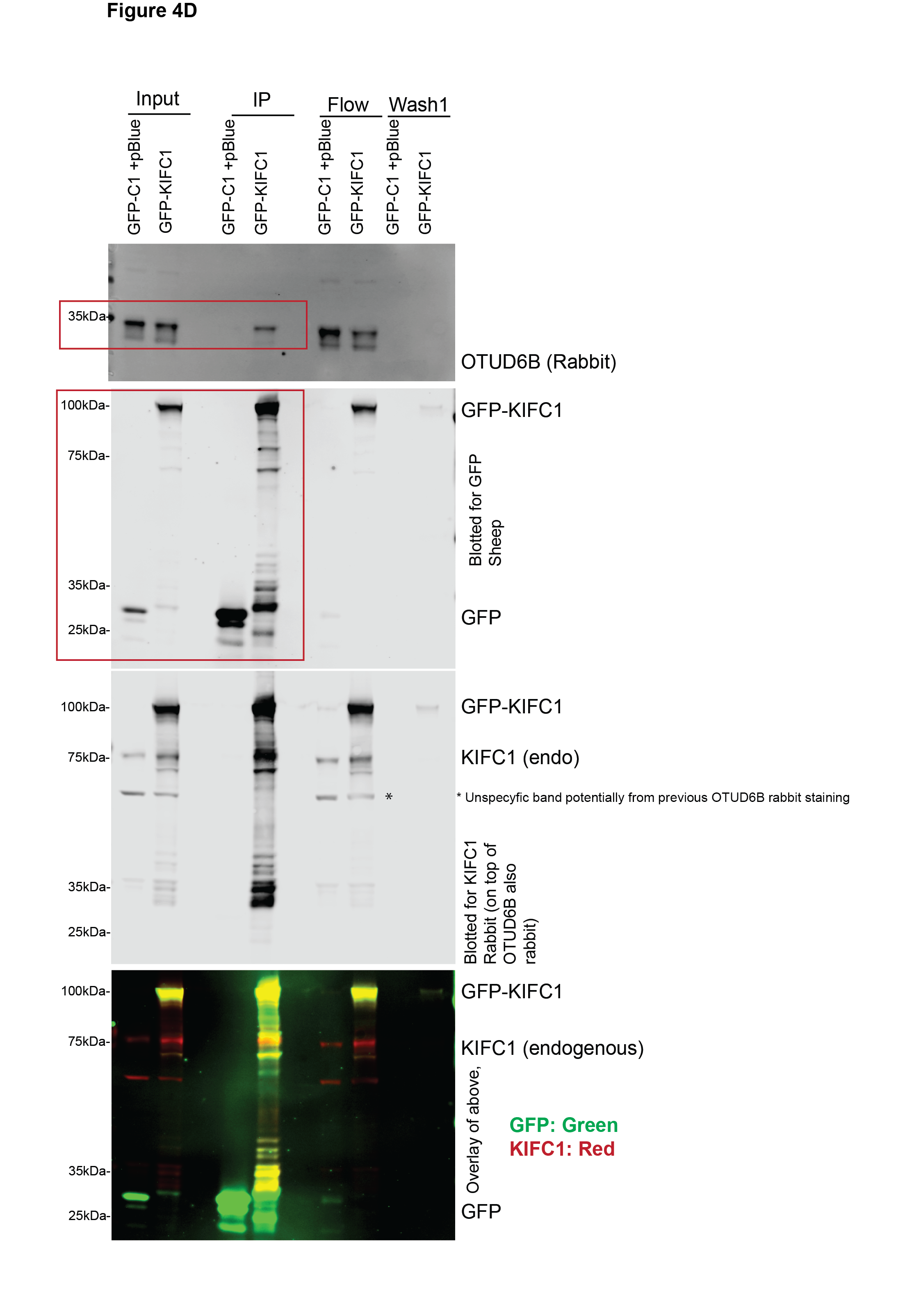

Supplement: Supplementary file 12 — Source data Fig. 4 [file 44319_2024_361_MOESM12_ESM.zip › Figure 4/EMBOR-2023-58722__Figure 4G/Figure 4G - IP.png]

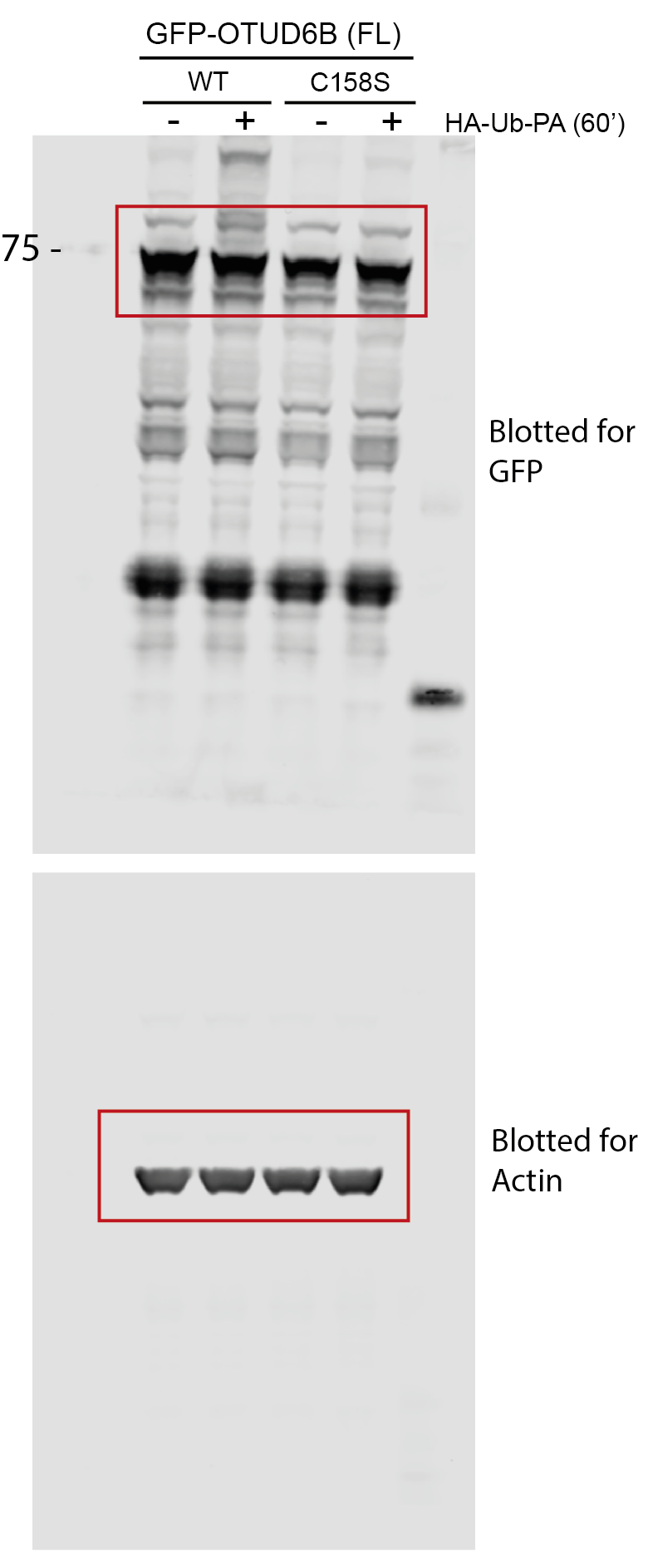

Supplement: Supplementary file 13 — Source data Fig. 5 [file 44319_2024_361_MOESM13_ESM.zip › Figure 5/Figure 5B/Figure 5B.png]

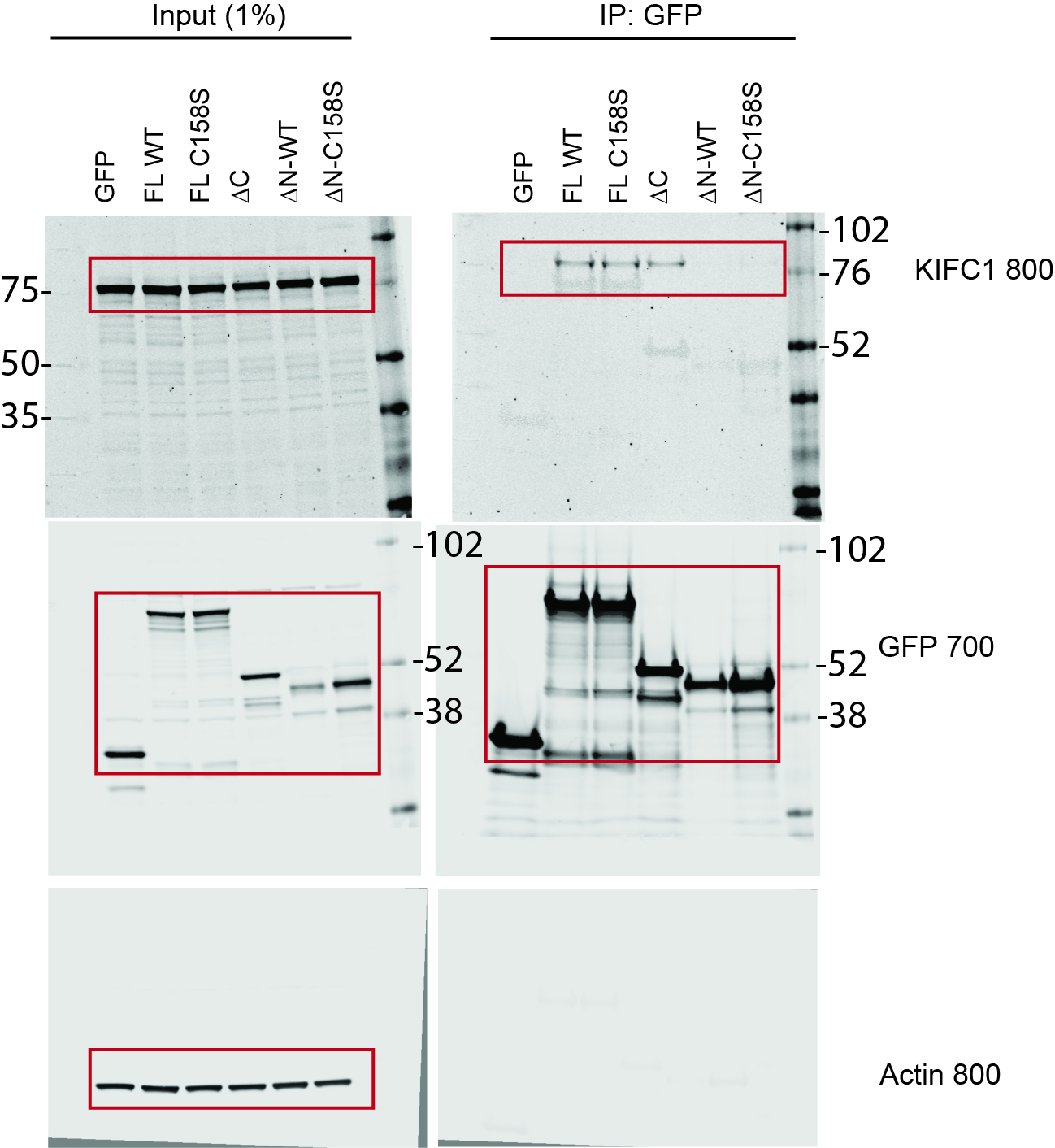

Supplement: Supplementary file 13 — Source data Fig. 5 [file 44319_2024_361_MOESM13_ESM.zip › Figure 5/Figure 5C/Figure 5C - IP.jpg]
