# Supplementary material for: OTUD6B regulates KIFC1-dependent centrosome clustering and breast cancer cell survival
Source: EMBO Rep. 2025 Jan 9;26(4):1003–35. doi: 10.1038/s44319-024-00361-w (PMC11850729; doi:10.1038/s44319-024-00361-w)
Supplement: Supplementary file 16 — Expanded View Figures [file 44319_2024_361_MOESM16_ESM.pdf]

Expanded View Figures

Figure EV1. Relationship between OTUD6B, centrosome amplification, KIFC1 expression and patient survival.

(**A**) Centrosome amplification in breast cancer and control cell lines. Cells were stained for α-tubulin (green), pericentrin (red), and DNA (DAPI, blue) and visualised using a Nikon Eclipse Ti fluorescent microscope. Centrosome number was scored in >100 mitotic cells per line. Scale bar 10 μm. (**B–D**) Correlation of KIFC1 and OTUD6B protein expression in a breast cell line panel. Representative immunoblot and quantitation normalised to actin shown relative to U2OS cell line, $n = 1$ experiment (**B**). Scatter plots comparing KIFC1 (**C**) or OTUD6B (**D**) with percentage centrosome amplification by two-tailed Pearson coefficient. MCF7 excluded from correlation as centrosome amplification low compared to other studies. (**E**) OTUD6B mRNA overexpression is an indicator of poor prognosis in breast cancer patients. Kaplan–Meier estimate of overall survival for all breast cancer patients stratified by high OTUD6B mRNA expression relative to diploid samples in the TCGA breast invasive carcinoma PanCancer Atlas; $n = 1084$ samples, $P = 0.00046$, long rank test. (**F**, **G**) KIFC1 and OTUD6B mRNA expression are most highly elevated in basal-like breast cancer in the TCGA PanCancer dataset. Patient data were stratified according to subtype; $n = 981$, $^{****}P \le 0.0001$ Kruskal–Wallis test with Dunn's multiple comparisons test. (**H**) Positive correlation of KIFC1 and OTUD6B protein expression in breast cancer patient samples classified as basal-like. Scatter plot comparing expression by mass spectrometry from the CPTAC breast invasive carcinoma dataset; $n = 23$, $^{*}P = 0.0452$ Pearson correlation.

 © The Author(s)

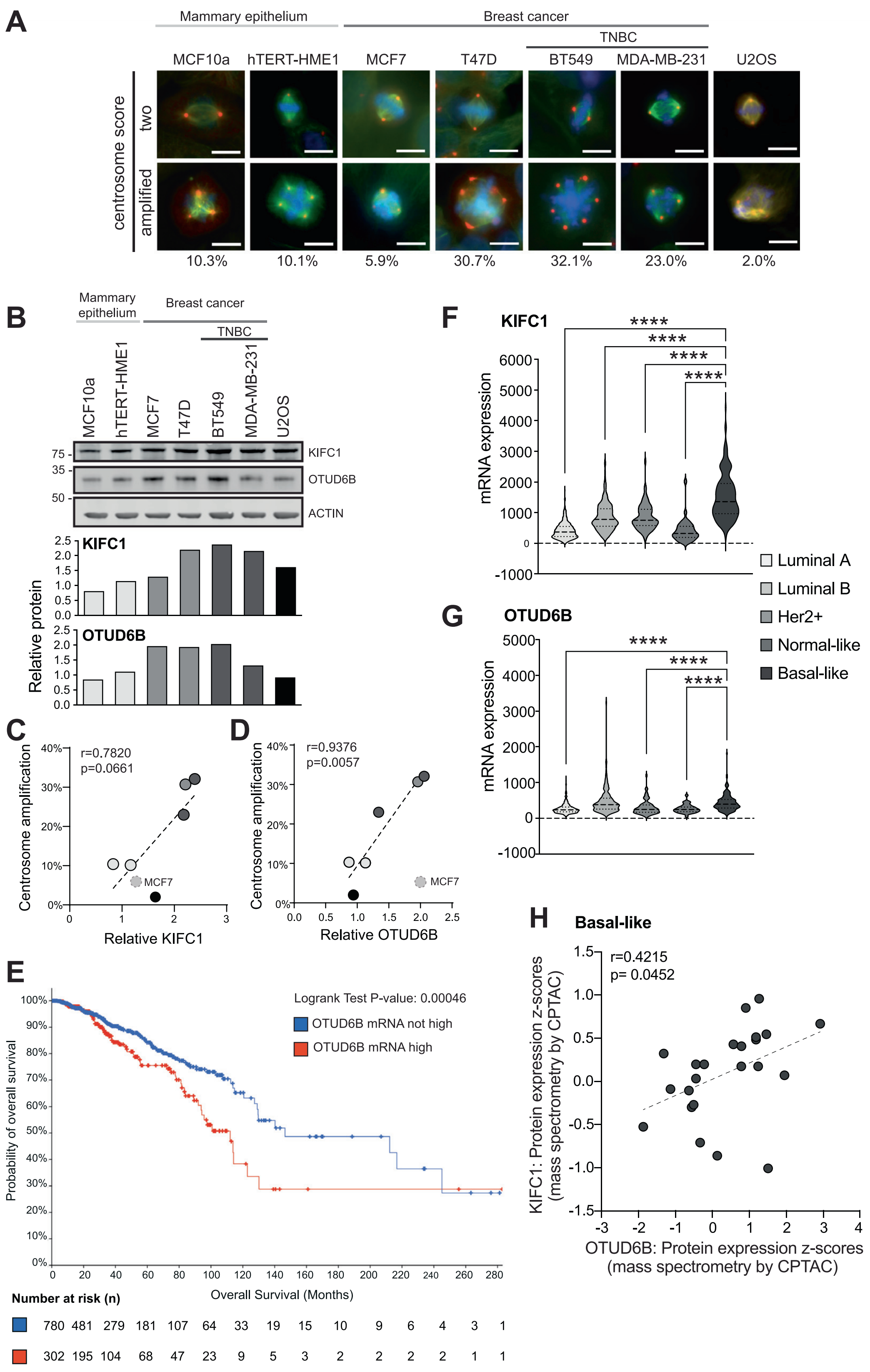

Survival plot summary

	Number of cases, Total	Number of Events	Median Months Overall (95% CI)
OTUD6B mRNA not high	780	94	146.50 (129.07 - NA)
OTUD6B mRNA high	302	57	112.08 (93.83 - NA)

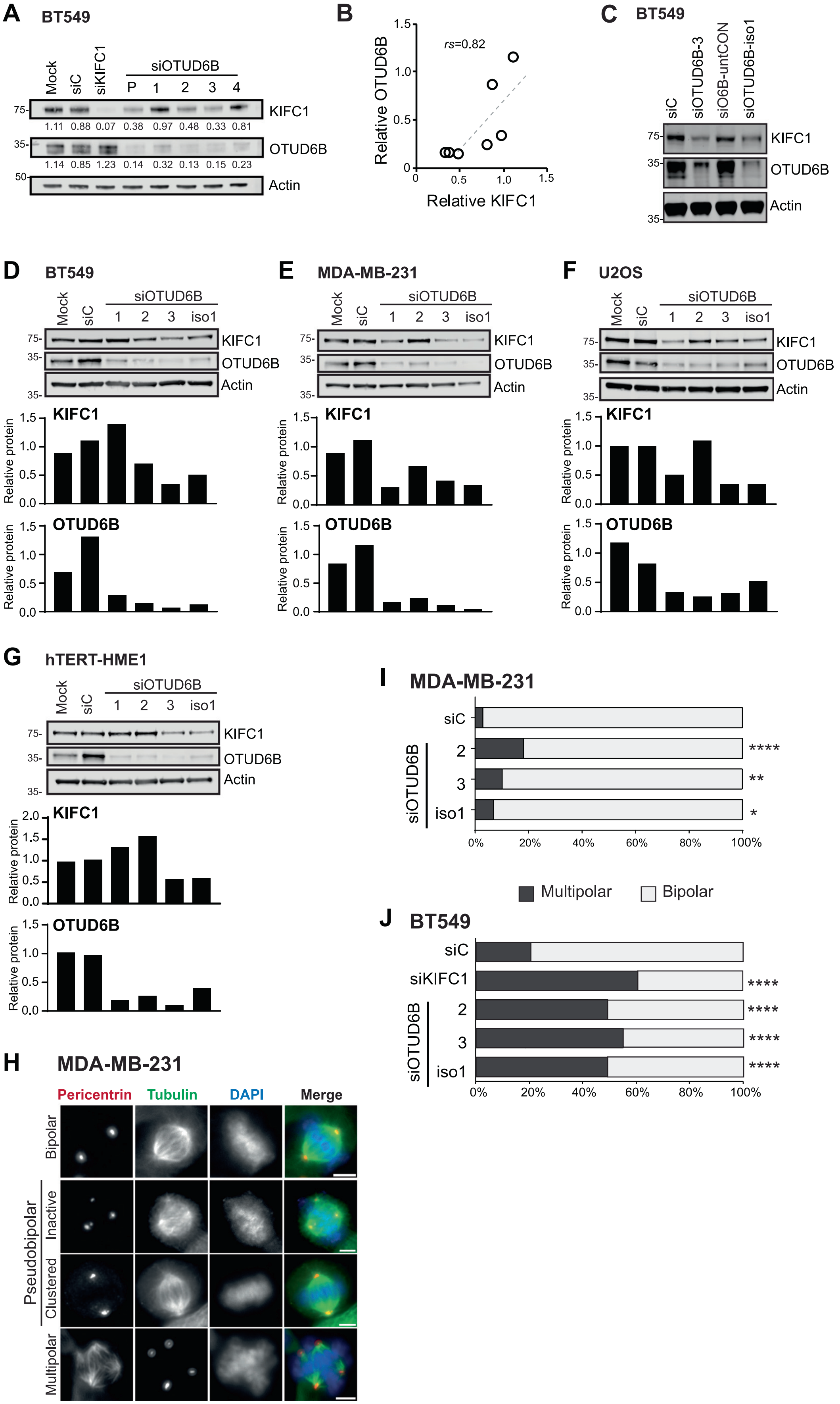
A BT549
Mock siC siKIFC1 siOTUD6B P 1 2 3 4
KIFC1
1.11 0.88 0.07 0.38 0.97 0.48 0.33 0.81
OTUD6B
1.14 0.85 1.23 0.14 0.32 0.13 0.15 0.23
Actin
B
rs=0.82
Relative OTUD6B
Relative KIFC1
C BT549
siC siOTUD6B-3 siO6B-untCON siOTUD6B-iso1
KIFC1
OTUD6B
Actin
D BT549
E MDA-MB-231
F U2OS
Mock siC siOTUD6B 1 2 3 iso1
KIFC1
OTUD6B
Actin
Relative protein
G hTERT-HME1
H MDA-MB-231
Pericentrin Tubulin DAPI Merge
Bipolar
Pseudobipolar
Inactive
Clustered
Multipolar
I MDA-MB-231
siC
siOTUD6B
2 ****
3 **
iso1 *
0% 20% 40% 60% 80% 100%
Multipolar Bipolar
J BT549
siC
siKIFC1 ****
2 ****
3 ****
iso1 ****

Figure EV2. OTUD6B knockdown reduces KIFC1 expression in various cell lines and increases multipolar spindles in breast cancer cell lines.

(**A–G**) Cells were transfected with 10 nM OTUD6B or control siRNAs and analysed 72 h post-transfection; protein levels normalised to actin and mean of the controls, one experiment shown. BT549 (**A–D**) showing correlation between OTUD6B and KIFC1 across samples (**B**). An siRNA specifically targeting the major OTUD6B isoform_1 (iso1) but not an untranscribed sequence 5′ of the OTUD6B gene (siO6B_untCON) reduces KIFC1 (**C**). OTUD6B depletion also reduces KIFC1 levels in MDA-MB-231 (**E**), U2OS (**F**) and hTERT-HME1 (**G**). (**H–J**) Three OTUD6B siRNAs induce multipolar spindles in TNBC cell lines with centrosome amplification. Cells were co-stained for centrosomes (pericentrin), spindle (tubulin) and DNA (DAPI); scale bar 10 µm (**H**). >100 metaphase cells were scored in each condition in MDA-MB-231 from $n = 1$ experiment, $^{****}P \leq 0.0001$, $^{**}P = 0.0027$ and $^{*}P = 0.0119$ (**I**), or BT549 cells across $n = 3$ biological replicates, $^{****}P \leq 0.0001$ (**J**) by One-sided Fisher's exact test compared to siC.

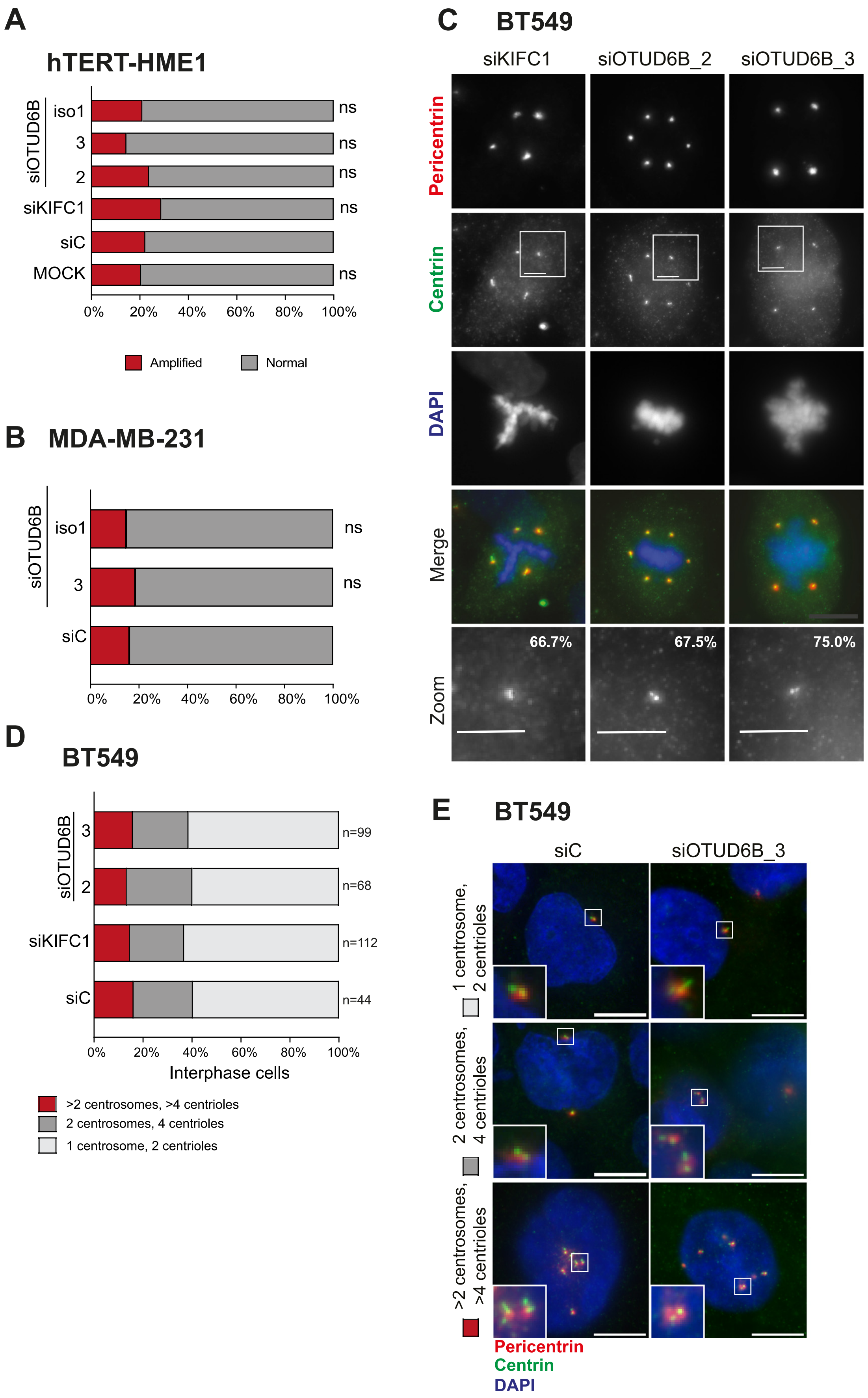
A
hTERT-HME1
siOTUD6B
iso1
3
2
siKIFC1
siC
MOCK
ns
ns
ns
ns
ns
0%
20%
40%
60%
80%
100%
Amplified
Normal
B
MDA-MB-231
siOTUD6B
iso1
3
siC
ns
ns
0%
20%
40%
60%
80%
100%
C
BT549
siKIFC1
siOTUD6B_2
siOTUD6B_3
Pericentrin
Centrin
DAPI
Merge
Zoom
66.7%
67.5%
75.0%
D
BT549
siOTUD6B
3
2
siKIFC1
siC
n=99
n=68
n=112
n=44
0%
20%
40%
60%
80%
100%
Interphase cells
>2 centrosomes, >4 centrioles
2 centrosomes, 4 centrioles
1 centrosome, 2 centrioles
E
BT549
siC
siOTUD6B_3
1 centrosome, 2 centrioles
2 centrosomes, 4 centrioles
>2 centrosomes, >4 centrioles
Pericentrin
Centrin
DAPI

 © The Author(s)

Figure EV3. OTUD6B depletion mimics the centrosome de-clustering phenotype of KIFC1 depletion without altering centrosome number.

(**A**, **B**) OTUD6B depletion does not induce centrosome amplification. The hTERT-HME1 breast epithelial cell line (**A**) or the MDA-MB-231 TNBC cell line (**B**) were transfected with 10 nM siRNA for 72 h, stained with tubulin and pericentrin and imaged with Nikon Eclipse Ti fluorescent microscope. >100 cells per condition were scored from $n = 3$ biological replicates (**A**) or 1 biological replicate (**B**); ns, not significant by One-sided Fisher's exact test compared to siC. (**C**) OTUD6B depletion mimics centrosome de-clustering. BT549 cells were transfected with 40 nM siRNA for 72 h then co-stained for pericentrin, centrin and DNA (DAPI), $n = 1$ experiment. Z-stacks of individual mitotic cells were acquired with a Nikon Eclipse Ti fluorescent microscope, (>15 mitotic cells per condition) and images presented as maximum-intensity projections. Insets show representative centrin foci; the percentage of multipolar spindles where all pericentrin foci contain at least two centrin foci is indicated. Scale bars, 10 μm. (**D**, **E**) OTUD6B depletion does not change centriole or centrosome number in interphase cells and does not induce centriole splitting. Experiment as described in (**C**) with >43 interphase cells imaged and scored for centriole and centrosome number (*n* indicated on graph), scale bar 5 μm.

© The Author(s)

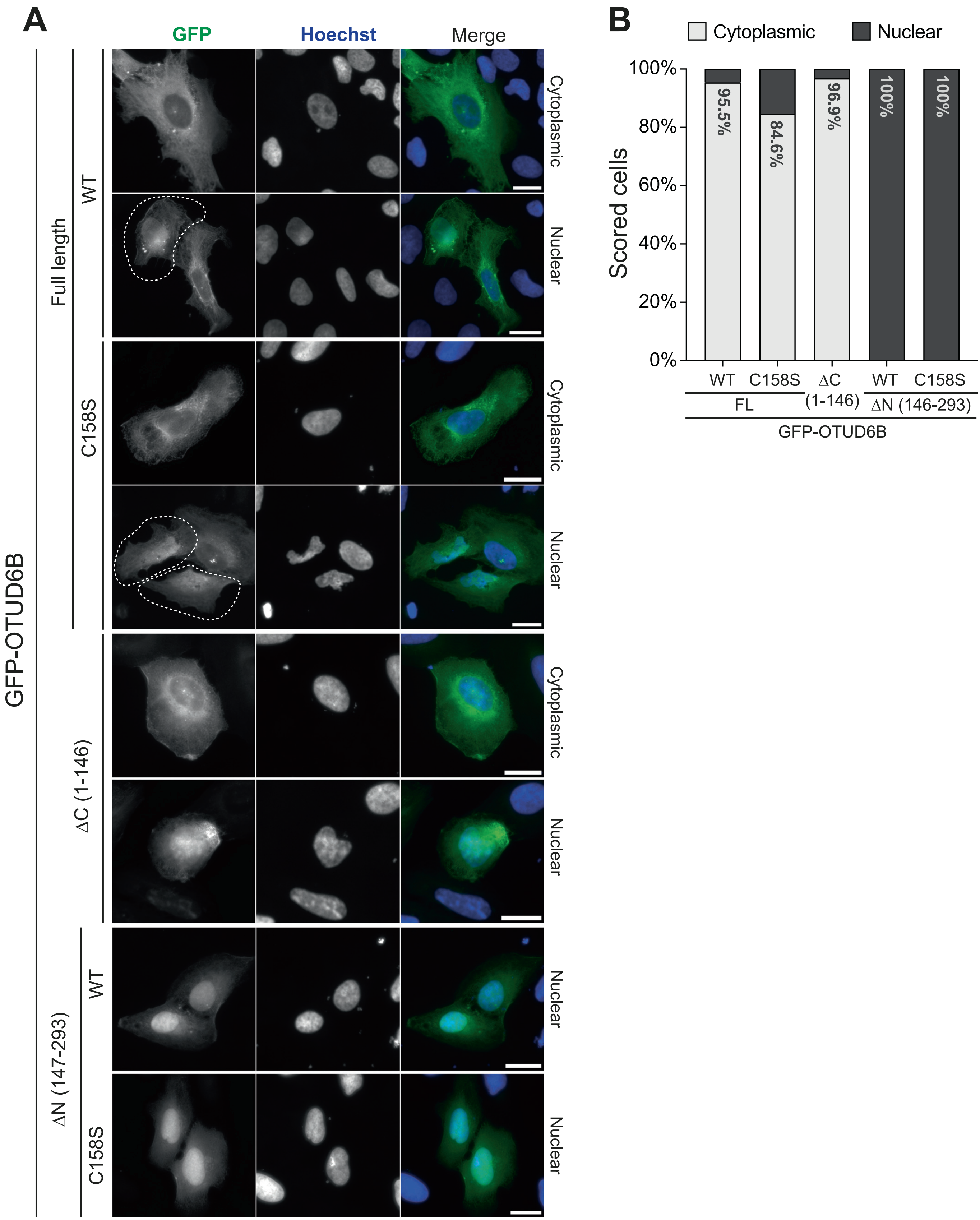
A
GFP
Hoechst
Merge
GFP-OTUD6B
Full length
WT
C158S
ΔC (1-146)
ΔN (147-293)
WT
C158S
Cytoplasmic
Nuclear
Cytoplasmic
Nuclear
Cytoplasmic
Nuclear
Nuclear
Nuclear
B
Cytoplasmic
Nuclear
Scored cells
100%
80%
60%
40%
20%
0%
95.5%
84.6%
96.9%
100%
100%
WT
C158S
ΔC (1-146)
WT
C158S
FL
ΔN (146-293)
GFP-OTUD6B

 © The Author(s)

Figure EV4. The N-terminus is required for OTUD6B nuclear exclusion.

(**A**, **B**) U2OS cells were transfected with plasmids for 48 h then stained for KIFC1, pericentrin and DNA (Hoechst). Images were acquired with a Nikon Eclipse Ti fluorescent microscope. Representative images of cytoplasmic or nuclear distribution observed in cells transfected with each construct; scale bar 10 µm; dotted lines indicate cells scored as nuclear in the field shown (**A**). >100 interphase cells were scored per condition from one experiment (**B**).

© The Author(s)

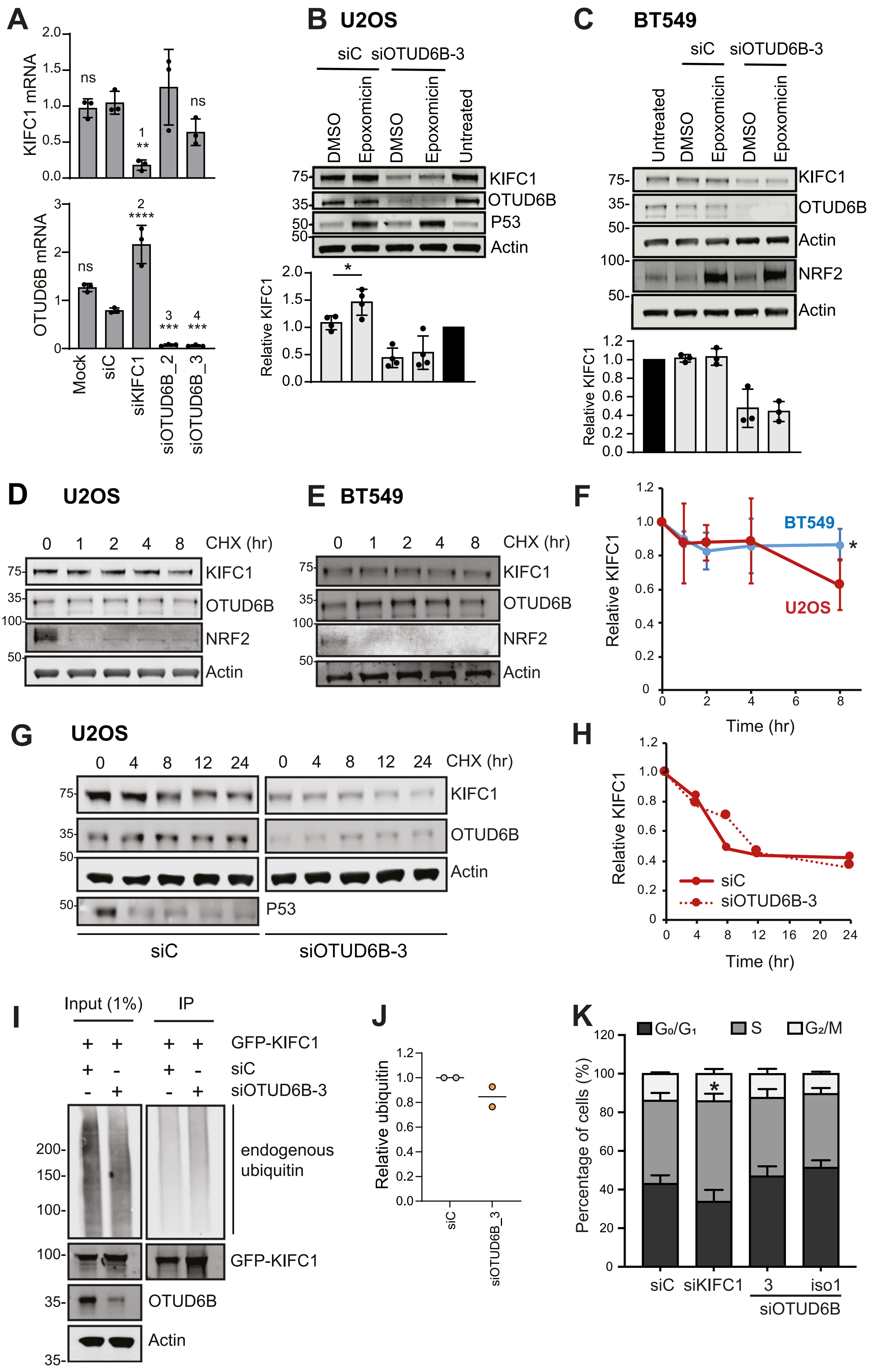
A
KIFC1 mRNA
ns
ns
1 **
OTUD6B mRNA
ns
2 ****
3 ***
4 ***
Mock
siC
siKIFC1
siOTUD6B_2
siOTUD6B_3
B U2OS
siC siOTUD6B-3
DMSO
Epoxomicin
DMSO
Epoxomicin
Untreated
KIFC1
OTUD6B
P53
Actin
Relative KIFC1
C BT549
siC siOTUD6B-3
Untreated
DMSO
Epoxomicin
DMSO
Epoxomicin
KIFC1
OTUD6B
Actin
NRF2
Actin
Relative KIFC1
D U2OS
CHX (hr)
KIFC1
OTUD6B
NRF2
Actin
E BT549
CHX (hr)
KIFC1
OTUD6B
NRF2
Actin
F
Relative KIFC1
BT549
U2OS
Time (hr)
G U2OS
CHX (hr)
KIFC1
OTUD6B
Actin
P53
siC
siOTUD6B-3
H
Relative KIFC1
siC
siOTUD6B-3
Time (hr)
I
Input (1%)
IP
GFP-KIFC1
siC
siOTUD6B-3
endogenous ubiquitin
GFP-KIFC1
OTUD6B
Actin
J
Relative ubiquitin
siC
siOTUD6B_3
K
G0/G1
S
G2/M
Percentage of cells (%)
siC
siKIFC1
3
iso1
siOTUD6B

Figure EV5. OTUD6B depletion does not affect KIFC1 transcription or global protein stability in asynchronous cells.

(**A**) OTUD6B depletion does not significantly alter KIFC1 transcription. BT549 cells were transfected with 10 nM siRNA for 72 h. Expression by qRT-PCR normalised to ACTB and GAPDH, shown relative to mean of controls; mean for $n = 3$ biological replicates, error bars SD, [1]$^{**}P = 0.0087$, [2]$^{****}P \leq 0.0001$, [3]$^{***}P = 0.0025$ and [4]$^{***}P = 0.0022$, compared to siC by one-way ANOVA with Dunnett post-hoc test. (**B**, **C**) U2OS (**B**) or BT549 (**C**) cells were transfected with 10 nM siRNA for 72 h and treated with 50 nM epoxomicin or DMSO for the final 6 h. Representative immunoblot (top) and mean values for KIFC1 expression normalised to actin and shown relative to the untreated sample for $n = 4$ (**B**) or $n = 3$ (**C**) biological replicates (below); error bars SD, $^{*}P = 0.0314$, compared to DMSO by two-tailed *t* test. (**D–F**) Comparison of KIFC1 and OTUD6B half-lives in U2OS (**D**) or BT549 (**E**) cell lines treated with 10 µg/ml cycloheximide (CHX). Mean expression relative to 0 h for $n = 3$ biological replicates (**F**), error bars SD, $^{*}P = 0.0416$, one-tailed t-test. (**G-H**) OTUD6B depletion does not reduce KIFC1 stability in asynchronous cells. U2OS cells were transfected with siRNA for 72 h, prior to CHX addition and immunoblotting (**G**), expression relative to 0 h, one experiment (**H**). (**I**, **J**) OTUD6B depletion does not increase KIFC1 ubiquitylation in asynchronous cells. U2OS cells were transfected with siRNAs for 72 h, and GFP-KIFC1 for 24 h then treated with 50n M epoxomicin for the final 6 h before immunoprecipitation with GFP nanobeads, $n = 2$ biological replicates. Representative immunoblot (**I**) and quantification of ubiquitin smear normalised to the total amount of GFP-KIFC1 pulled down (**J**). (**K**) KIFC1 but not OTUD6B depletion increases the proportion of cells in S-phase. MDA-MB-231 cells were transfected with 10 nM siRNAs for 72 h, stained with 7-AAD and analysed by flow cytometry; error bars SD of mean for n = 3 biological replicates; $^{*}P = 0.0459$ compared to siC by one-way ANOVA with Dunnett post-hoc test.

© The Author(s)
